# Two New Bioactive α-Pyrones from *Hypericum japonicum*

**DOI:** 10.3390/molecules21040515

**Published:** 2016-04-19

**Authors:** Linzhen Hu, Zhenzhen Wang, Jinwen Zhang, Yuanyuan Lu, Kaiping Wang, Yongbo Xue, Yu Zhang, Yonghui Zhang

**Affiliations:** 1 Union Hospital Affiliated to Tongji Medical College, Huazhong University of Science and Technology, Wuhan 430022, China; hlz198@126.com; 2 Hubei Key Laboratory of Natural Medicinal Chemistry and Resource Evaluation, School of Pharmacy, Tongji Medical College, Huazhong University of Science and Technology, Wuhan 430030, China; wzz75283@163.com (Z.W.); wkpzcq@163.com (K.W.); yongboxue@hust.edu.cn (Y.X.); 3 Tongji Hospital Affiliated to Tongji Medical College, Huazhong University of Science and Technology, Wuhan 430030, China; tjzhangjinwen@163.com (J.Z.); yuanyuanlu2016@163.com (Y.L.)

**Keywords:** *Hypericum japonicum*, pyrones, Kaposi’s sarcoma associated herpes virus

## Abstract

*Hypericum japonicum* (Guttiferae), a type of annual or perennial herb, has been historically applied to cure infectious hepatitis, acute and chronic hepatitis, gastrointestinal disorder, and internal hemorrhage. In our successive studies on the genus *Hypericum*, two new α-pyrones termed japopyrones A and B (**1** and **2**) were isolated from *H. japonicum*. Their structures and absolute configurations were established by the comprehensive analyses of spectroscopic data, the application of the Single-crystal X-ray diffraction structural analysis, and the experimental electronic circular dichroism (ECD) spectra. Bioactivity screenings suggested that compound **2** possessed the potential inhibition efficacy on lytic replication of Kaposi’s sarcoma associated herpesvirus (KSHV) with an IC_50_ 29.46 μM and a selective index of higher than 6.79, respectively.

## 1. Introduction

**Figure 1:**
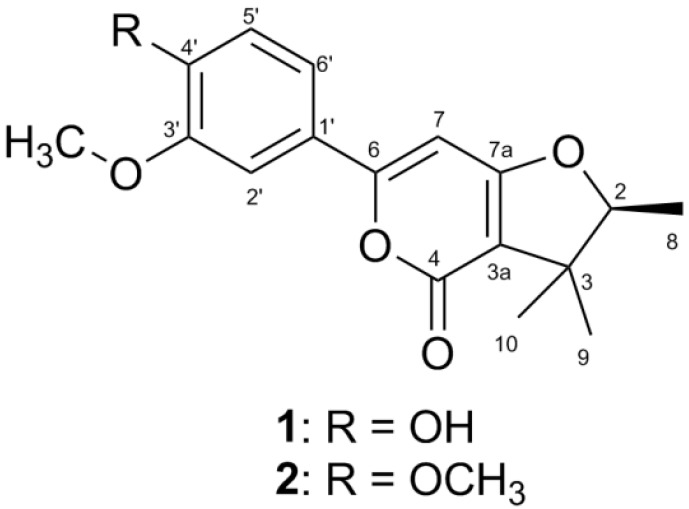
Structures of compounds **1** and **2**.

*Hypericum japonicum* (Guttiferae), a type of annual or perennial herb, is prosperously distributed from Liaoning and Shandong Provinces to the Southern Provinces of the Yangtze River in China [1]. As a type of Chinese traditional medicine, *H. japonicum* is mainly applied to cure infectious hepatitis, acute and chronic hepatitis, gastrointestinal disorder, and internal hemorrhage [2]. Furthermore, the extracts of *Hypericum* have been recorded as anti-depressant drugs in Europe and the Americas [1,3]. Currently, phytochemical investigations on this plant have led to the isolation of diverse chemical constituents such as xanthones [4], flavonoids [5], and phloroglucinols [6]. In our successive studies on the genus *Hypericum* [7,8,9], two new α-pyrones termed japopyrones A and B (**1** and **2**) (Figure 1), were obtained from the air-dried aerial parts of *H. japonicum*. Previous reports showed that α-pyrones served as bacterial signaling entities in the insect pathogen *Photorhabdus* [10], while some γ*-*pyrones exhibited coagulant activities *in vitro* [3]. Herein, the isolation, the structure elucidation, and the anti-Kaposi’s sarcoma associated herpesvirus (KSHV) activities of compounds **1** and **2** are described.

## 2. Results

The air-dried aerial parts of *H. japonicum* (10 kg) were soaked in 95% EtOH, which afforded a crude extract (800 g) under a vacuum evaporation. The crude extract was suspended in water and extracted sequentially with petroleum ether and CHCl_3_ for three times. The petroleum ether fraction (100 g) was chromatographed by silica gel column chromatography (silica gel CC), RP-18 Middle Pressure Liquid Chromatography (MPLC), and High Performance Liquid Chromatography (HPLC) to yield two new α-pyrones (**1** and **2**) as shown in Figure 1, which were termed as japopyrones A and B, respectively.

Japopyrone A (**1**), colorless crystal, ${\left[\alpha \right]}_{D}^{20}$ −55.7 (c 0.06, CH_3_OH), has the molecular formula of C_17_H_18_O_5_, which was deduced by the HRESIMS positive peak at *m*/*z* 325.1082 ([M + Na]^+^, calcd as 325.1052) and ^13^C-NMR data. The analysis of IR spectrum implicated the characteristic functionalities with absorption bands at 3314 cm^−^^1^ (hydroxyl), 1697 cm^−^^1^ (conjugated ester carbonyl), and 1562 and 1512 cm^−^^1^ (phenyl). Compared the NMR data of **1** with the reported ones of saropyrone [11], the difference between compound **1** and saropyrone is that a methoxyl function at C-3′ of **1** is replaced by a hydroxyl group in saropyrone. Coupled with the analysis of the HSQC spectrum, the ^1^H- and ^13^C-NMR data were unambiguous assigned as shown in Table 1. The ^1^H-NMR spectrum presented the signals of three methyls (δ_H_ 1.34, d, *J* = 6.6 Hz; 1.30, s; and 1.11, s), one methoxyl (δ_H_ 3.83, s), three aromatic protons (δ_H_ 7.35, s; 6.87, d, *J* = 8.0 Hz; and 7.33, d, *J* = 8.0 Hz), one proton (δ_H_ 6.94, s), and one methine proton (δ_H_ 4.61, q, *J* = 6.6 Hz). The ^13^C-NMR and DEPT 135 spectra showed 17 carbon signals which were eight quaternary carbon atoms (including one carbonyl, one aliphatic, and six aromatic/olefinic carbon atoms), five methines (including four aromatic/olefinic and one aliphatic carbon atom), three methyls, and one methoxyl (δ_C_ 55.8). The above analyses showed that compound **1** is a class of α-pyrones.

**Figure 2:**
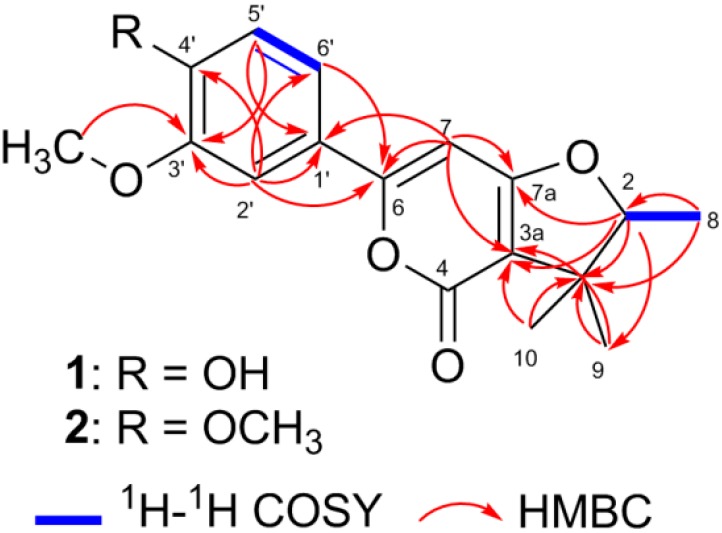
Key HMBC and ^1^H-^1^H COSY correlations of compounds **1** and **2**.

Detailed analyses of the HMBC and ^1^H-^1^H COSY spectra resulted in the structural connection of **1** (Figure 2). The HMBC spectrum exhibited the cross-peaks from H-2′ to C-1′, C-3′, C-4′, C-6′, and C-6, from H-5′ to C-1′ and C-3′, from H-6′ to C-6, and from C3′-OCH_3_ to C-3′, as well as a H-5′/H-6′ spin system of the ^1^H-^1^H COSY spectrum, which manifested the connection of a 1′,3′,4′-substituted benzene ring with an oxygen-bearing olefinic carbon viz. C-6′. Furthermore, HMBC cross-peaks detected from H-7 to C-1′, C-6, C-3a, and C-7a, implied the position of the olefinic double bond (∆^6,7^). In addition, HMBC correlations were observed from Me-8 to C-2 and C-3, from Me-9 and Me-10 to C-3 and C-3a, and from H-2 to C-3, C-3a, and C-7a, together with an H-2/H-8 spin system of the ^1^H-^1^H COSY experiments, which indicated the location of Me-8, Me-9, and Me-10 at the furan ring and confirmed the fusion between furan and pyrone rings via C-7a and C-3a.

**Figure 3:**
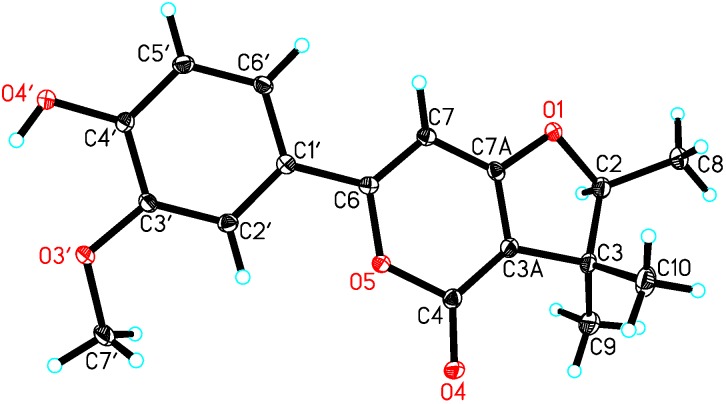
X- ray structure of **1**.

The absolute configuration of compound **1** was determined by a single-crystal X-ray diffraction structural analysis. Using the program SHELXL-2014/7, the structure solution and the refinement were achieved, which unequivocally established that the chiral characteristic of **1** was 2*S*. The X-ray ORTEP drawing of **1** was shown in Figure 3 (Flack’s parameter 0.01(4), CCDC 1456415).

Japopyrone B (**2**) was isolated as white amorphous powder with ${\left[\alpha \right]}_{D}^{20}$ −87.0 (*c* 0.06, CH_3_OH). Its molecular formula C_18_H_20_O_5_ was deduced by the positive pseudomolecular ion peak at *m*/*z* 317.1382 ([M + H]^+^, calcd as 317.1389) from a HRESIMS experiment and ^13^C-NMR data. A careful comparison of the 1D NMR data between **1** and **2** (Table 1) showed that the main differentiation between **1** and **2** were the presence of a hydroxyl group in 1 instead of a methoxyl group in **2** at C-4′. The key 2D correlations of compounds **2** were identical with **1** (Figure 2).

**Figure 4:**
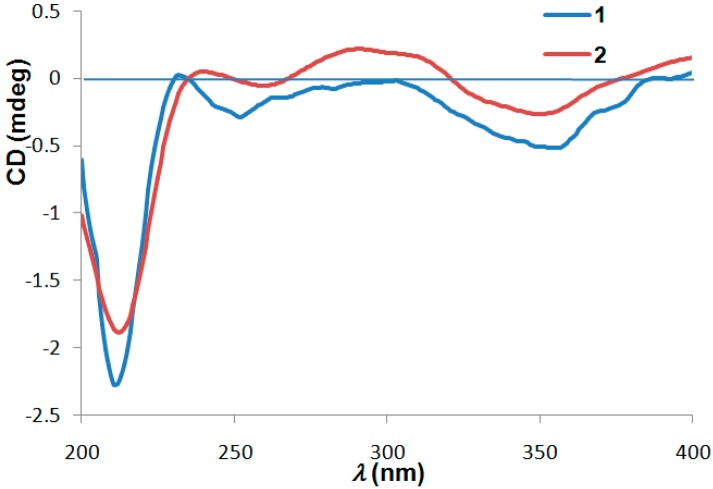
Experimental electronic circular dichroism (ECD) spectra of **1** and **2** (in CH_3_OH).

With the aid of experimental ECD spectra, the absolute sterochemistry of **2** was confirmed to be 2*S*, which was secured by the similar Cotton effects between **1** and **2** (Figure 4). Moreover, the levorotatory optical activities of compounds **1** and **2** also implied their coincident chiral characteristics.

Natural products have provided a rich resource for the discovery of new drugs, innovative therapeutic agents, and lead structures [12]. In our studious research towards the discovery for new lead compounds and useful bioactivities from Chinese traditional herbs, several bioactivity screenings such as cytotoxicity assays against five human cancer cell lines (HL-60, SMMC-7721, A-549, MCF-7, and SW480), inhibitory activities on NO production, and inhibitory activities on β-site amyloid precursor protein cleaving enzyme 1 (BACE1), were carried out for compounds **1** and **2**. Unfortunately, both **1** and **2** exhibited inert activities with IC_50_ > 40 µM for cytotoxicity assays, IC_50_ > 25 µM for NO production inhibition assay, and IC_50_ > 40 µM for BACE1 inhibition assay, respectively.

**Figure 5:**
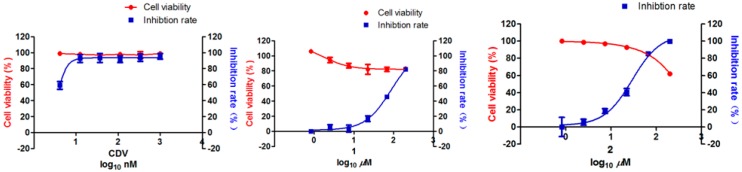
The effects of positive control cidofovir (CDV), **1** and **2** on human iSLK.219 cells viabilities and on lytic replication of KSHV infecting Vero cells were measured *in vitro*.

Human gamma herpes viruses such as Kaposi’s sarcoma-associated herpes virus (KSHV) is a type of pathogenic virus related to Kaposi’s sarcoma, like epidemic KS, posttransplant KS, multicentric Castleman’s disease, and primary effusion lymphoma [13,14]. Anti-infection towards lytic replication of KSHV plays a pivotal role as decreased a risk of KS, which were evidenced in cases of AIDS-associated epidemic KS patients [15,16]. In our exhaustive study to explore bioactivities of metabolites, an inhibition assay on lytic replication of KSHV was investigated for compounds **1** and **2** referring to the previous experiments [17]. The results (Table 2 and Figure 5) suggested that compound **2** had a potential efficacy with IC_50_ 29.46 µM of inhibition towards 12-*O*-tetradecanoylphorbol-13-acetate (TPA)-induced lytic replication of KSHV, with the value of CC_50_ higher than 200 µM, which means that the selective index is higher than 6.79. Meanwhile, compound **1** exhibited a moderate inhibition with IC_50_ 85.34 µM and CC_50_ higher than 200 µM. The details of the dose-dependent manner were shown in Figure S1, Supplementary Materials. More elaborate procedures of anti-KSHV assay were also stated in Supplementary Materials.

## 3. Materials and Methods

### 3.1. General Experiments

The following apparatuses were applied to acquire isolations and physical parameters of compounds **1** and **2**. Silica gel H (160–200 mesh, Shanghai Xibao Biological Technology Co. Ltd, Shanghai, China) was used in column chromatography. ODS (50 μm, Merck Co. Ltd., Darmstadt, Germany) and Sephadex LH-20 (GE Healthcare Bio-Sciences AB, Uppsala, Sweden) were taken as packing materials. High Performance Liquid Chromatography (HPLC) were carried out via a LC 3050 Analysis of HPLC system (CXTH, Beijing, China) assembled with an UV 3000 detector and a semi-preparative column (5 μm, 10 × 250 mm, YMC^®^ XB-C_18_). High-resolution electrospray ionization mass spectra (HRESIMS) were performed using a Thermo Fisher LC-LTQ-Orbitrap XL spectrometer (Thermo Fisher Scientific Inc., Waltham, MA, USA). UV and IR spectra data were recorded by a Varian Cary 50 (Varian Medical Systems, Salt Lake City, UT, USA) and Bruker Vertex 70 (Brucker Corporation, Karlsruhe, Germany) apparatuses. A Bruker AM-600/400 spectrometer (Brucker Corporation) was implemented to afford NMR spectra. The chemical shifts of ^1^H- and ^13^C-NMR were referenced to the solvent peaks for DMSO-*d*_6_ at δ_H_ 2.50 and δ_C_ 39.5 and methanol-*d*_4_ at δ_H_ 3.31 and δ_C_ 49.2. Optical rotation values were recorded by a Perkin-Elmer 341 polarimeter (Perkin Elmer Inc., Waltham, MA, USA).

### 3.2. Plant Material

The air-dried aerial parts of *H. japonicum* were collected in November 2013 at Da-Bie Mountain area of Hubei Province, China and identified by Prof. Jianping Wang, School of Pharmacy, Tongji Medical College, Huazhong University of Science and Technology. A voucher sample (No. 2013-1111) has been deposited in the Herbarium of Hubei Key Laboratory of Natural Medicinal Chemistry and Resource Evaluation, School of Pharmacy, Tongji Medical College, Huazhong University of Science and Technology, Wuhan, China.

### 3.3. Extraction and Isolation

The air-dried aerial parts of *H. japonicum* (10 kg) were extracted four times with 95% aqueous EtOH at 40 °C, which furnished extracts (800 g) under vacuum evaporation. The extracts were suspended in the water and sequentially extracted with petroleum ether and trichloromethane. TLC analyses were used to guide the next isolation project. The petroleum ether extracts (100 g) were subjected to silica gel CC via a gradient elution (petroleum ether–acetone, 100:1–1:1) to yield 10 fractions (Fr. 1–Fr. 10). Based on the TLC analysis, Fr. 8 was chosen and further repurified by normal-phase silica gel CC, reversed-phase silica gel CC, and Sephadex LH-20 to afford five subfractions (Fr. 8.1–Fr. 8.5). Finally, Fr. 8.3 was subjected to semi-preparative HPLC (CH_3_OH-H_2_O 35%) to obtain **1** (4.2 mg) and **2** (4.5 mg).

*Japopyrone A* (**1**): colorless crystal; ${\left[\alpha \right]}_{D}^{20}$ −55.7 (*c* 0.06, CH_3_OH); UV (CH_3_OH) λ_max_ (log *ε*) 213 (4.33), 235 (3.10) 351 (3.21) nm; IR (KBr) ν_max_ 3314, 2966, 2933, 1697, 1621, 1582, 1562, 1512 cm^−1^; ECD λ_max_ (∆ε) 211 (−0.79) , 252 (−0.10), 355 (−0.18) nm; ^1^H- and ^13^C-NMR data, see Table 1; HRESIMS: *m*/*z* 325.1082 [M + Na]^+^ (calcd for C_17_H_18_O_5_Na, 325.1052).

Japopyrone B (**2**): white amorphous powder; ${\left[\alpha \right]}_{D}^{20}$ −87.0 (*c* 0.06, CH_3_OH); UV (CH_3_OH) λ_max_ (log ε) 214 (4.41), 344 (4.29) nm; IR (KBr) ν_max_ 2967, 1725, 1564, 1515 cm^−1^; ECD λ_max_ (∆ε) 212 (−4.28), 240 (+0.11), 291 (+0.50), 350 (−0.60) nm; ^1^H- and ^13^C-NMR data, see Table 1; HRESIMS: *m*/*z* 317.1382 [M + H]^+^ (calcd for C_18_H_21_O_5_, 317.1389).

Single-crystal data for japopyrone A (**1**): C_17_H_18_O_5_, M = 302.31, *a* = 9.1711(2) Å, *b* = 11.4036(3) Å, *c* = 14.6711(4) Å, α = 90°, β = 90°, γ = 90°, *V* = 1534.36(7) Å^3^, *T* = 100(2) K, space group P212121, *Z* = 4, μ (CuKα) = 0.798 mm^−1^, 16,146 reflections measured, 2847 independent reflections (*R*_int_ = 0.0364). The final *R*_1_ values were 0.0293 (*I* > 2σ(*I*)). The final *wR*(*F*_2_) values were 0.0780 (*I* > 2*σ*(*I*)). The final *R*_1_ values were 0.0293 (all data). The final *wR*(*F*_2_) values were 0.0780 (all data). The goodness of fit on *F*_2_ was 1.102. Flack parameter = 0.01(4).

The crystallographic data of japopyrone A (**1**): CCDC 1456415 contains the supplementary crystallographic data for this paper. These data can be obtained free of charge from the Cambridge Crystallographic Data Centre via www.ccdc.cam.ac.uk/data_request/cif.

## 4. Conclusions 

Two new bioactive α-pyrones, namely, japopyrones A (**1**) and B (**2**), were isolated from the aerial parts of *Hypericum japonicum*. The absolute configurations were determined by the analyses of the extensive spectra including HRESIMS, NMR, UV, and IR spectra, the application of the Single-crystal X-ray diffraction structural analysis, and the experimental electronic circular dichroism (ECD) spectra. Bioactivity screenings suggested that compound **2** had potential inhibition efficacy on lytic replication of KSHV with an IC_50_ of 29.46 μM and the selective index being higher than 6.79. 

## Figures and Tables

**Table 1:** ^1^H-NMR (400 MHz) and ^13^C-NMR (100 MHz) Spectral Data of Compounds **1** and **2** (δ in ppm, *J* in Hz; ^a^ in DMSO-*d*_6_ and ^b^ in methanol-*d*_4_).

NO.	1 ^a^		2 ^b^	
	δ_H_ (ppm)	δ_C_ (ppm)	δ_H_ (ppm)	δ_C_ (ppm)
2	4.61q (*J* = 6.6 Hz)	91.4	4.64q (*J* = 6.6 Hz)	93.9
3		42.1		43.9
4		159.7		163.5
6		162.7		164.8
7	6.94s	91.6	6.77s	93.7
8	1.34d (*J* = 6.6 Hz)	14.4	1.42d (*J* = 6.6 Hz)	14.9
9	1.30s	25.1	1.40s	25.9
10	1.11s	20.1	1.21s	20.6
1′		122.4		125.5
2′	7.35s	109.4	7.43d (*J* = 2.1 Hz)	110.2
3′		149.7		150.9
4′		147.9		153.5
5′	6.87d (*J* = 8.0 Hz)	115.7	7.05d (*J* = 8.6 Hz)	112.9
6′	7.33d (*J* = 8.0 Hz)	119.4	7.50dd (*J* = 8.5, 2.2 Hz)	120.8
3a		107.2		109.6
7a		169.7		172.8
3′-OCH_3_	3.83s	55.8	3.90s	56.7
4′-OCH_3_			3.89s	56.6

^a^: in DMSO-*d*_6_ ; ^b^: in methanol-*d*_4_.

**Table 2:** Anti-Kaposi’s sarcoma associated herpesvirus (KSHV) activities of positive control cidofovir (CDV), **1**, and **2** (µM).

Compounds	CC_50_	IC_50_	Selective Index (CC_50_/IC_50_)
CDV	>1	<0.004	>250
**1**	>200	85.34	>2.34
**2**	>200	29.46	>6.79

## Supplementary Materials

The following are available online at: http://www.mdpi.com/1420-3049/21/4/515/s1, anti-KSHV assay, HRESIMS, NMR, UV, and IR spectra of compounds **1** and **2**.

## Abbreviations

The following abbreviations are used in this manuscript:

*H. japonicum*: *Hypericum japonicum*
ECD: electronic circular dichroism
HRESIMS: High-resolution electrospray ionization mass spectra
CC: column chromatography
HPLC: High Performance Liquid Chromatography
BACE1: β-site amyloid precursor protein cleaving enzyme 1
KSHV: Kaposi’s sarcoma associated herpes virus
TPA: 12-*O*-tetradecanoylphorbol-13-acetate
CDV: cidofovir
EtOH: ethanol
