# Supplementary material for: Two New Bioactive α-Pyrones from Hypericum japonicum
Source: Molecules. 2016 Apr 19;21(4):515. doi: 10.3390/molecules21040515 (PMC6272854; doi:10.3390/molecules21040515)
Supplement: Supplementary file 1 [file molecules-21-00515-s001.pdf]

# Supplementary Materials: Two New Bioactive $\alpha$-Pyrones from *Hypericum Japonicum*

**Linzhen Hu, Zhenzhen Wang, Jinwen Zhang, Yuanyuan Lu, Kaiping Wang, Yongbo Xue, Yu Zhang and Yonghui Zhang**

# Content

**Anti-KSHV assay** ........ S2

**Figure S1**. HRESIMS spectrum of japopyrone A (**1**). ........ S2

**Figure S2**. $^1$H-NMR spectrum of japopyrone A (**1**) (in DMSO-$d_6$). ........ S3

**Figure S3**. $^{13}$C-NMR and DEPT 135 spectra of japopyrone A (**1**) (in DMSO-$d_6$)........ S3

**Figure S4**. HSQC spectrum of japopyrone A (**1**) (in DMSO-$d_6$). ........ S3

**Figure S5**. HMBC spectrum of japopyrone A (**1**) (in DMSO-$d_6$)........ S4

**Figure S6**. $^1$H-$^1$H COSY spectrum of japopyrone A (**1**) (in DMSO-$d_6$). ........ S4

**Figure S7**. UV spectrum of japopyrone A (**1**). ........ S5

**Figure S8**. IR spectrum of japopyrone A (**1**). ........ S5

**Figure S9**. HRESIMS spectrum of japopyrone B (**2**). ........ S6

**Figure S10**. HRESIMS spectrum of japopyrone B (**2**). ........ S6

**Figure S11**. $^1$H-NMR spectrum of japopyrone B (**2**) (in methanol-$d_4$). ........ S7

**Figure S12**. HSQC spectrum of japopyrone B (**2**) (in methanol-$d_4$)........ S7

**Figure S13**. HMBC spectrum of japopyrone B (**2**) (in methanol-$d_4$)........ S7

**Figure S14**. $^1$H–$^1$H COSY spectrum of japopyrone B (**2**) (in methanol-$d_4$). ........ S8

**Figure S15**. UV spectrum of japopyrone B (**2**). ........ S8

**Figure S16**. IR spectrum of japopyrone B (**2**). ........ S9

**References** ........ S9

**Anti-KSHV assay**

Human iSLK.219 cells were used to determine the antiviral activity of desired compounds against KSHV. The cells harbor the rKSHV.219 virus that encodes green fluorescent protein (GFP) under control of the elongation factor 1$\alpha$ promoter (EF-1$\alpha$) and lytic replication of KSHV was reactivated with 1 μg/mL doxycycline (Dox) (Beyotime) and 1.2 mM sodium butyrate (NaB) (Sigama) [1,2]. Cells grown to 70% confluence 96-well tissue culture plates were treated with indicated concentrations of compounds in present of Dox and NaB. Then cell viability was assessed at 48 h post drugs-induction by AlamarBlue® Cell Viability Assay (Invitrogen) according to the manufacturer's protocol. The luminescent signal was measured using the Envison 2102 Multilabel Reader (Perkin Elmer, Waltham, MA, USA). The 50% cytotoxic concentration ($CC_{50}$) for each compound was calculated from these dose-response curves using Graphpad5.0 Prism. The results were presented as mean values with standard deviations ($n$ = 3) (shown in Figure S1).

The antiviral effects of compounds on KSHV were determined by infectivity assay as previously described [3]. The supernatants from iSLK.219-treated or untreated with the compounds in the presence of Dox and NaB were collected at 48 h. Then, the supernatants were used to infect the Vero cells seeded in a 96-well plate as previously reported using centrifugation at 1500× *g* for 60 min [4]. The supernatants were then removed and replaced with flash DMEM medium. At 48 h, the expression of GFP per well in Vero cells was detected and analyzed using the Operetta High-Content Screening System (HCS) (Perkin Elmer). Nine image fields per well were recorded by the automated microscope based HCS and the GFP intensity per well was calculated using the Harmony 3.5 software (Perkin Elmer). Data were normalized as the fold change compared to the DMSO control. The $IC_{50}$ (50% effective concentration) values correspond to compound concentrations required to reduce quantitative expression of the intensity of GFP by 50%. The results are presented as the mean values with standard deviations ($n$ = 3) (shown in Figure S1).

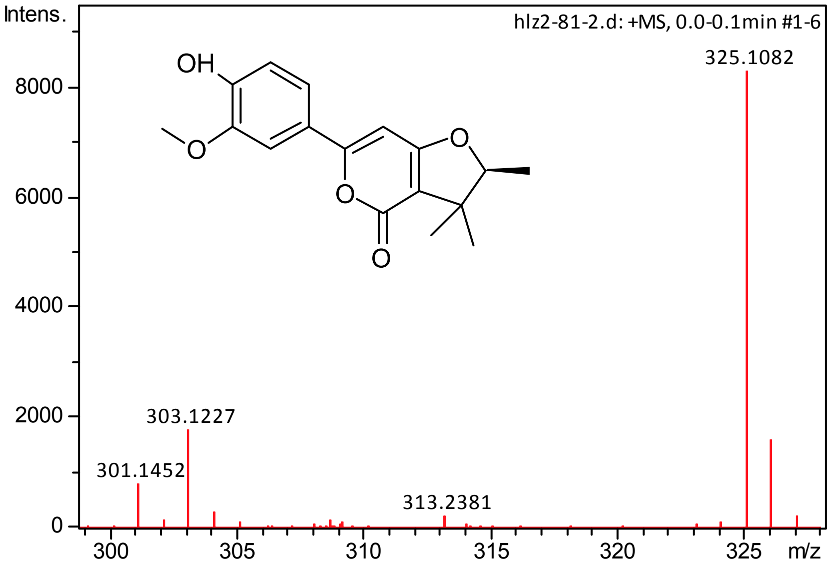


**Figure S1**. HRESIMS spectrum of japopyrone A (**1**).

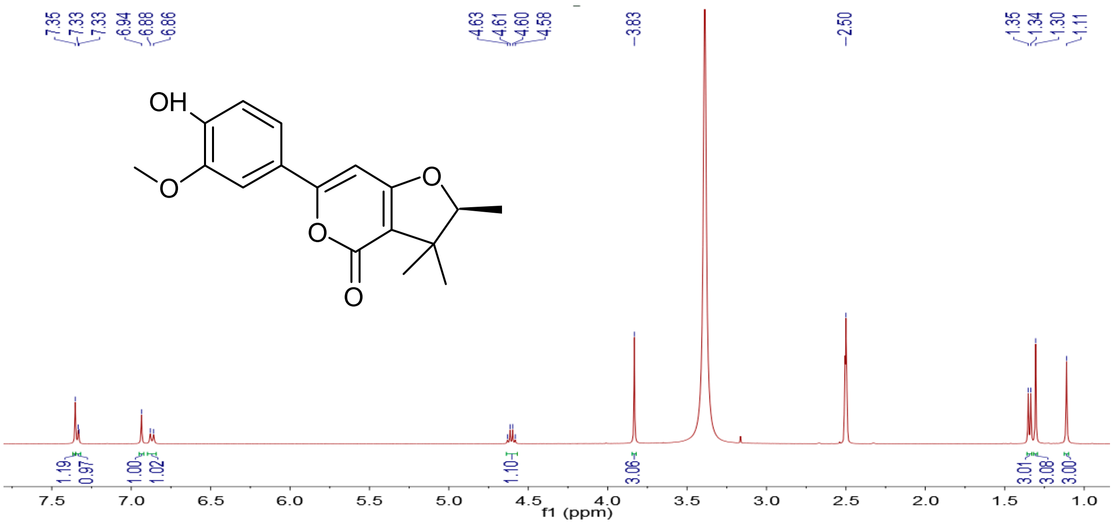


**Figure S2.** $^{1}$H-NMR spectrum of japopyrone A (**1**) (in DMSO-*d*6).

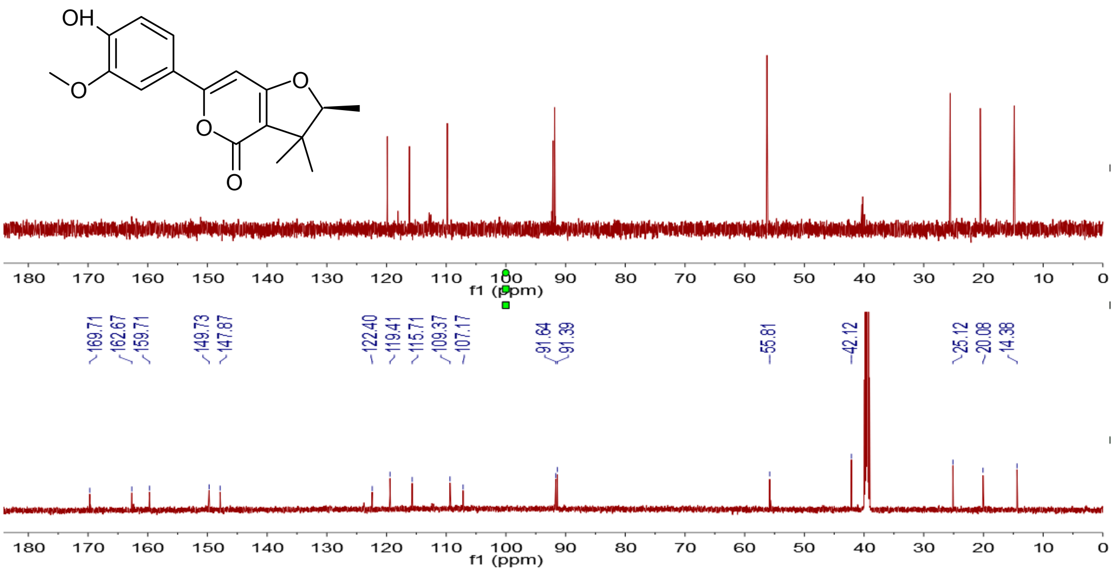


**Figure S3.** $^{13}$C-NMR and DEPT 135 spectra of japopyrone A (**1**) (in DMSO-*d*6).

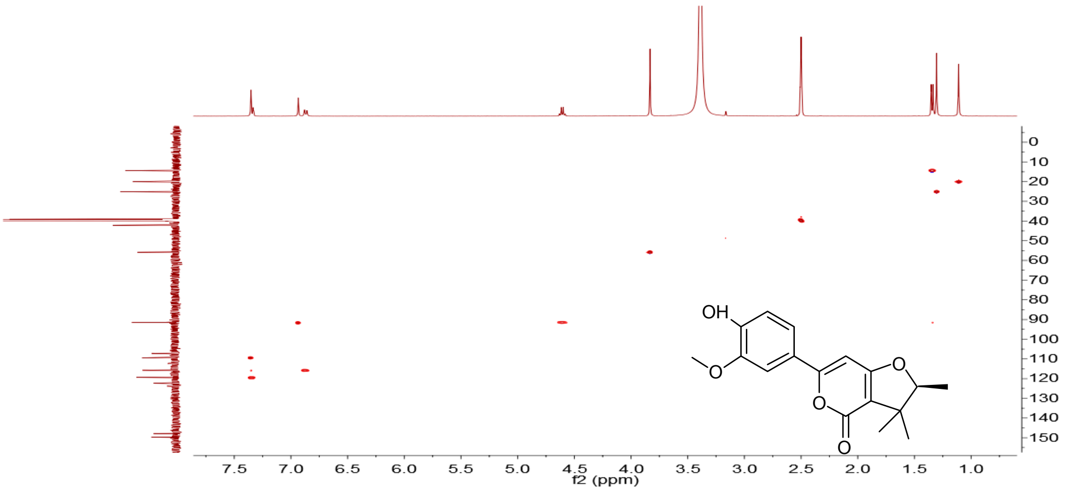


**Figure S4.** HSQC spectrum of japopyrone A (**1**) (in DMSO-*d*6).

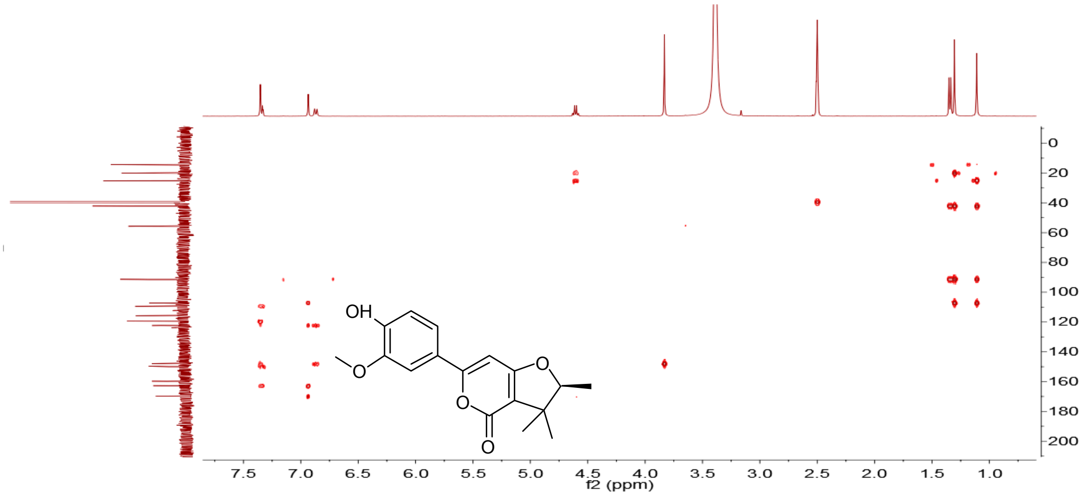


**Figure S5**. HMBC spectrum of japopyrone A (**1**) (in DMSO-$d_6$).

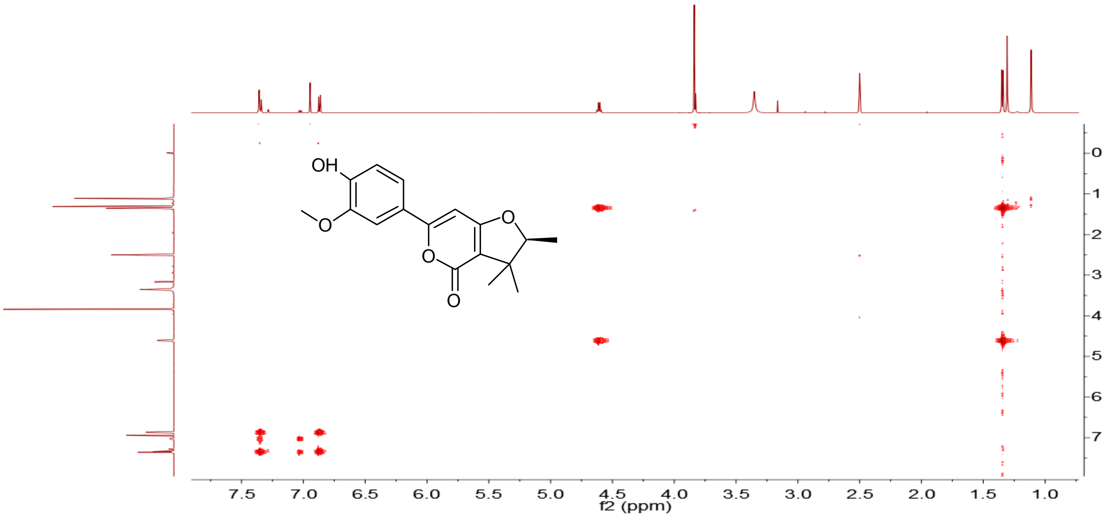


**Figure S6**. $^1$H–$^1$H COSY spectrum of japopyrone A (**1**) (in DMSO-$d_6$).

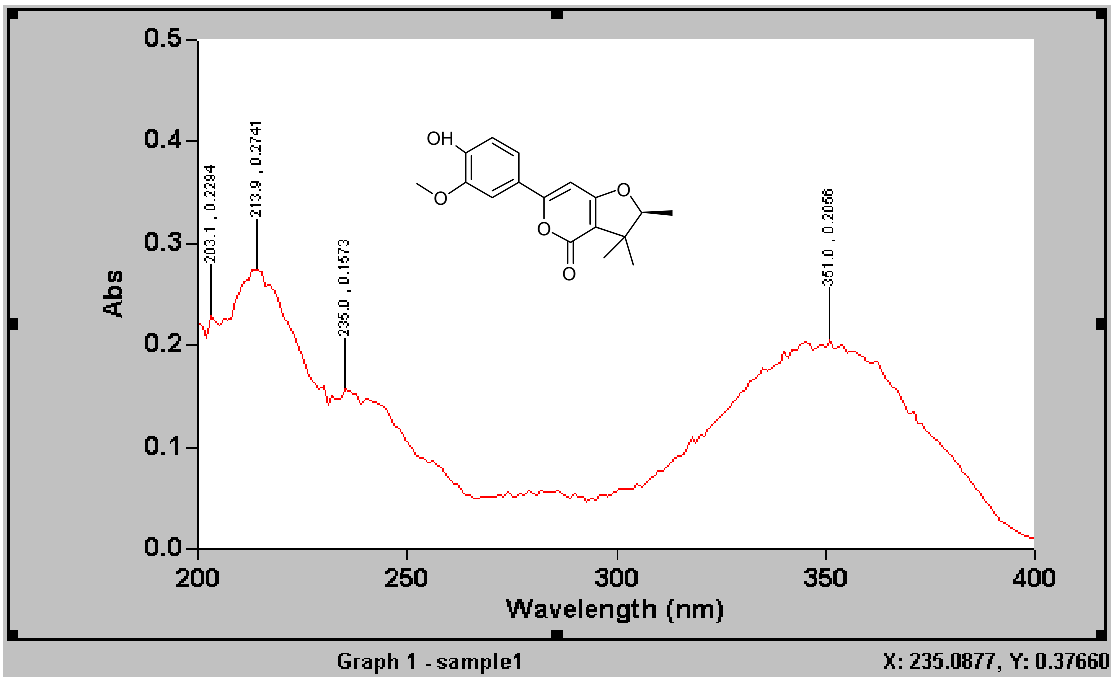


**Figure S7**. UV spectrum of japopyrone A (**1**).

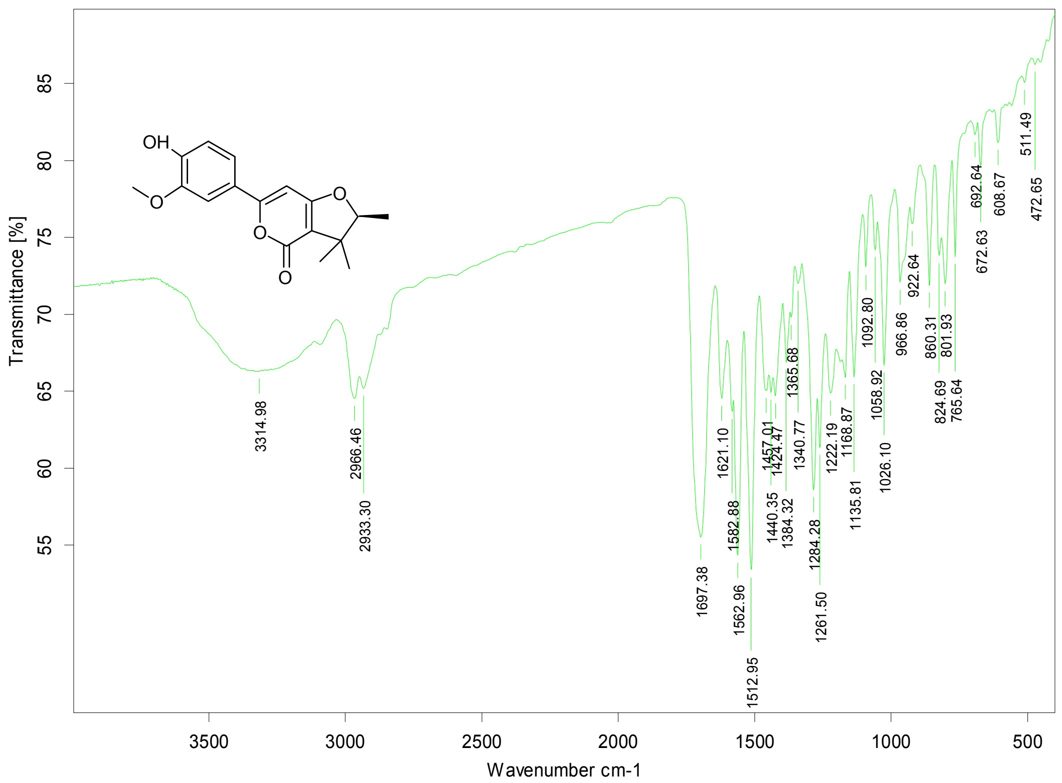


**Figure S8**. IR spectrum of japopyrone A (**1**).

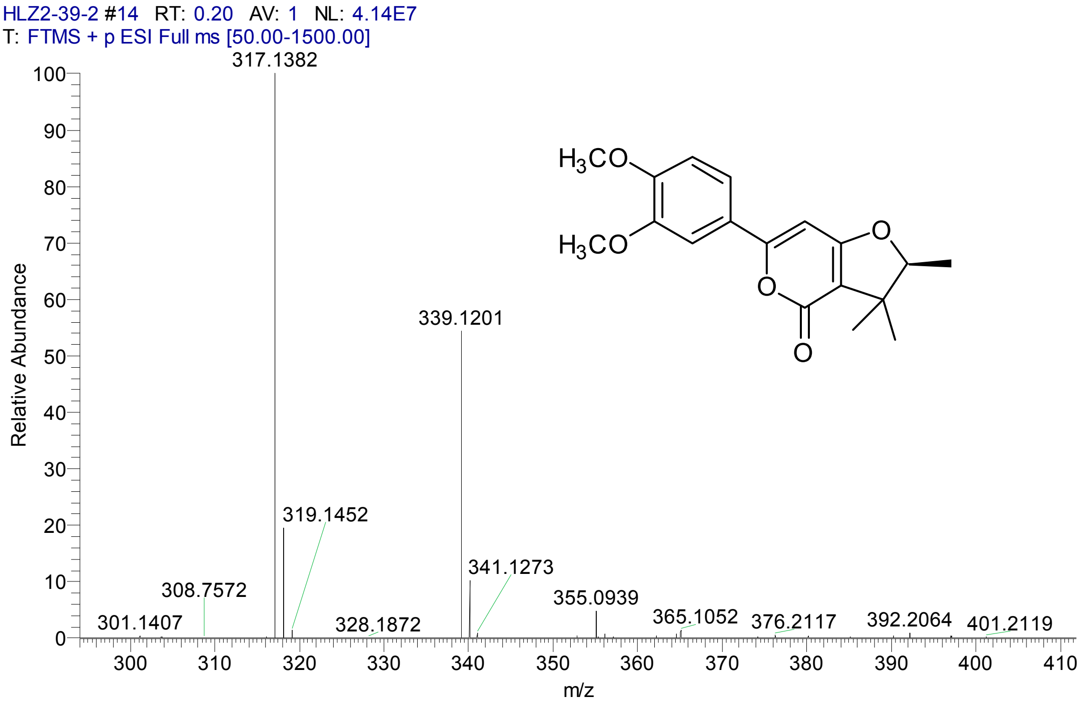


**Figure S9**. HRESIMS spectrum of japopyrone B (**2**).

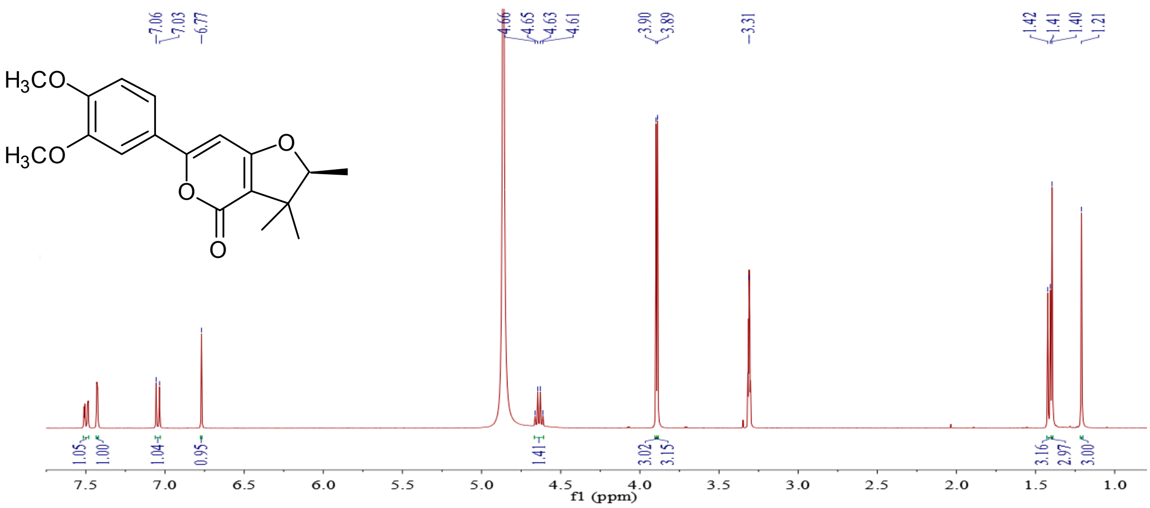


**Figure S10**. $^{1}$H-NMR spectrum of japopyrone B (**2**) (in methanol-*d*4).

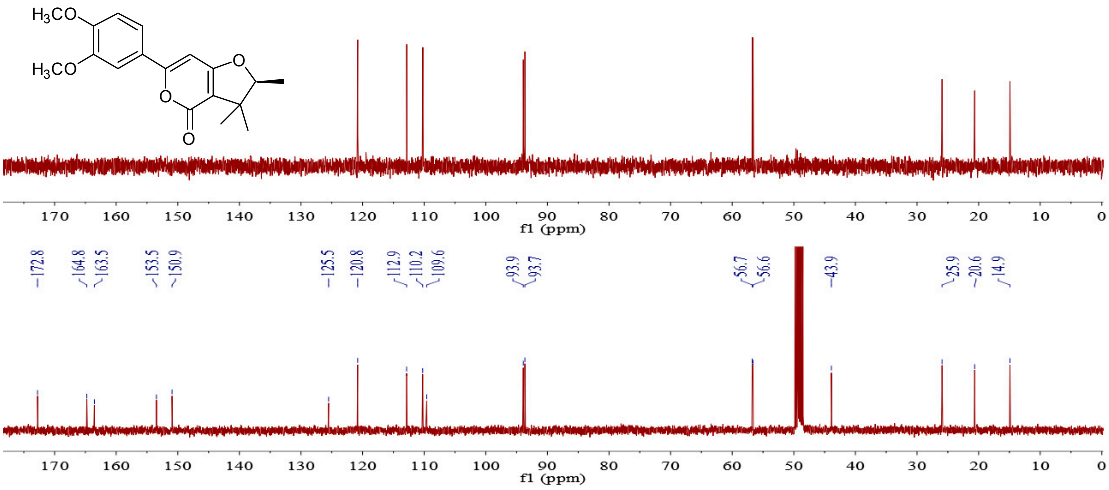


**Figure S11**. $^{13}$C-NMR and DEPT 135 spectra of japopyrone B (**2**) (in methanol-*d*4).

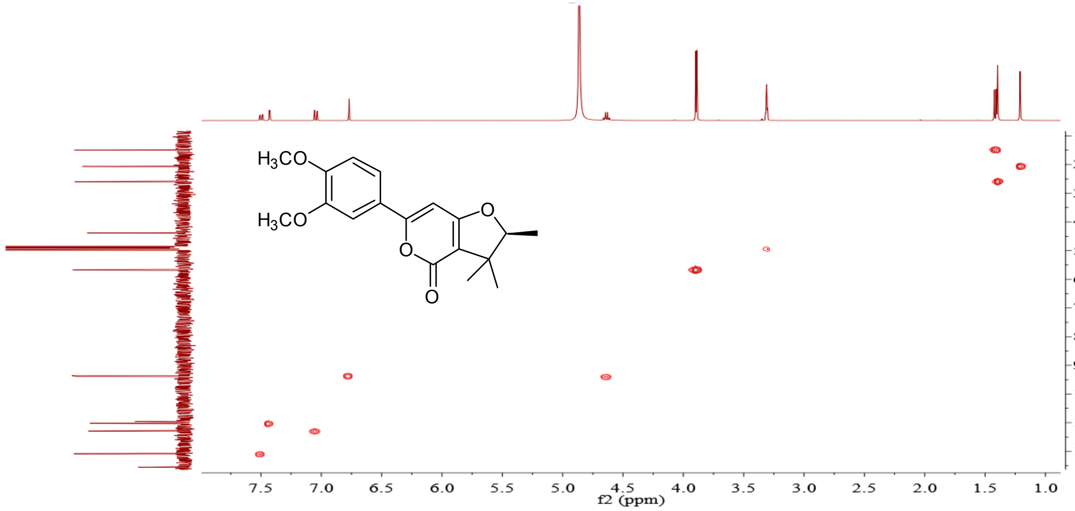


**Figure S12**. HSQC spectrum of japopyrone B (**2**) (in methanol-*d*4).

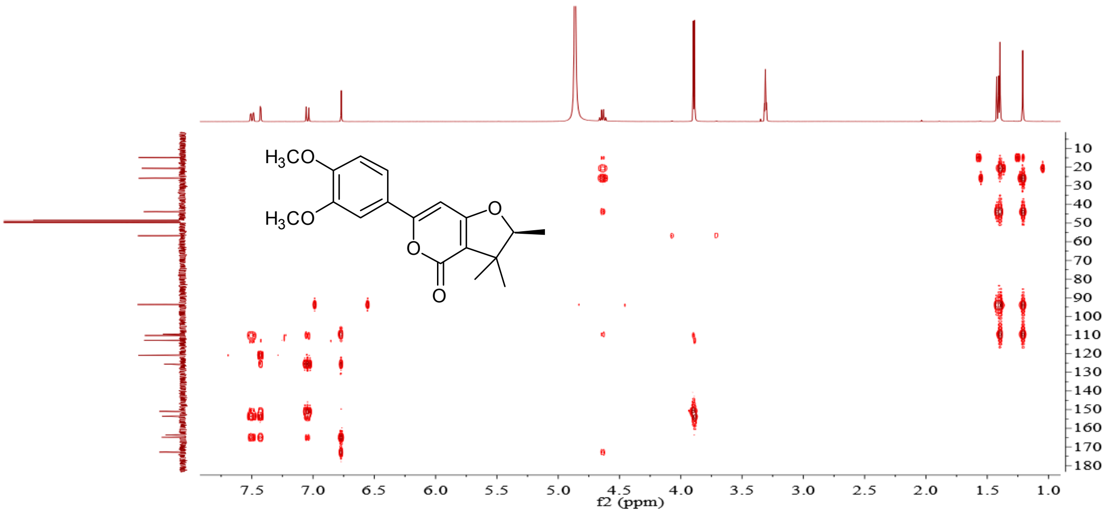


**Figure S13.** HMBC spectrum of japopyrone B (**2**) (in methanol-*d*4).

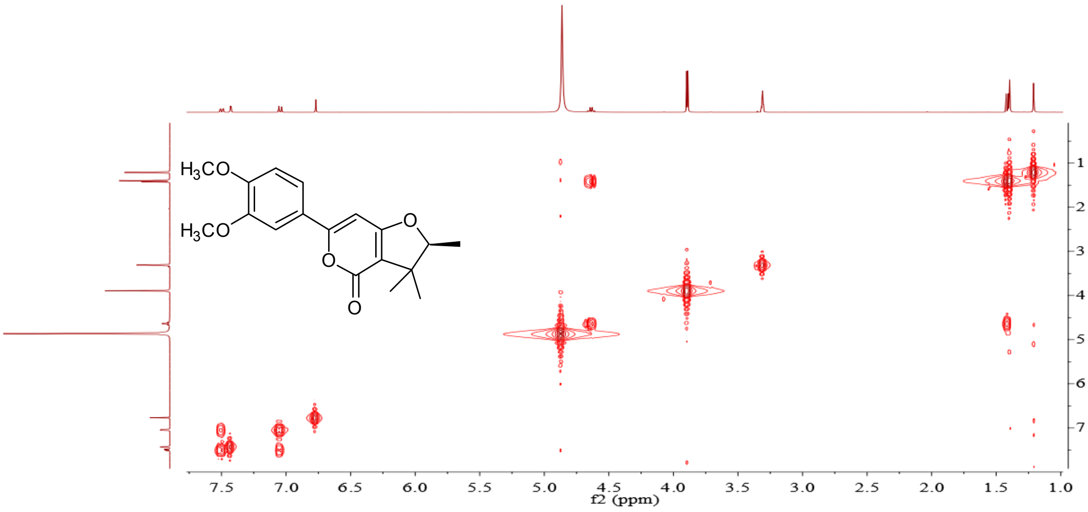


**Figure S14.** $^1$H-$^1$H COSY spectrum of japopyrone B (**2**) (in methanol-*d*4).

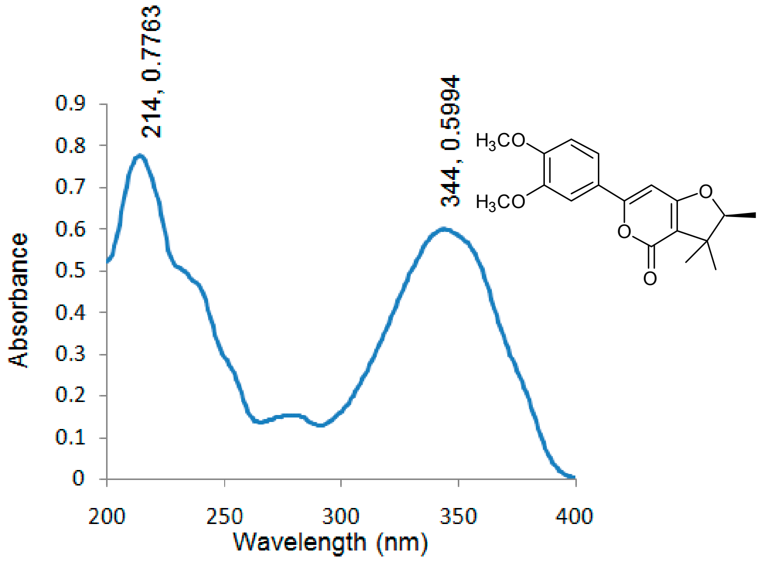


**Figure S15.** UV spectrum of japopyrone B (**2**).

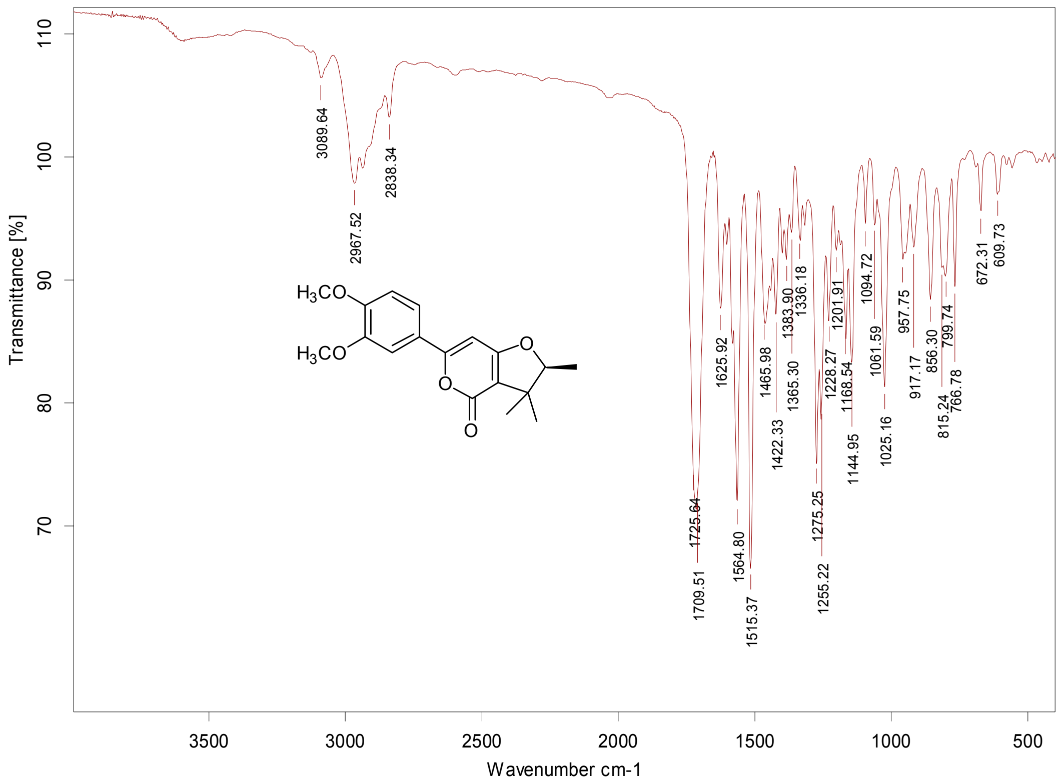


**Figure S16**. IR spectrum of japopyrone B (**2**).

## References


1 Myoung, J.; Ganem, D. Generation of a doxycycline-inducible KSHV producer cell line of endothelial origin: maintenance of tight latency with efficient reactivation upon induction. *J. Virol. Methods* **2011**, *174*, 12–21.

2 Vieira, J.; O'Hearn, P.M. Use of the red fluorescent protein as a marker of Kaposi's sarcoma-associated herpesvirus lytic gene expression. *Virology* **2004**, *325*, 225–40.

3 Chen, J.; Jiang, L.; Lan, K.; Chen, X., Celecoxib inhibits the lytic activation of Kaposi's Sarcoma-Associated Herpesvirus through down-regulation of RTA expression by inhibiting the activation of p38 MAPK. *Viruses* **2015**, *7*, 2268–2287.

4 Yoo, S.M.; Ahn, A.K.; Seo, T.; Hong, H.B.; Chung, M.A.; Jung, S.D.; Cho, H.; Lee, M.S. Centrifugal enhancement of Kaposi's sarcoma-associated virus infection of human endothelial cells *in vitro*. *J. Virol. Methods* **2008**, *154*, 160–1666.
